# Building primary health care from the ground up: family medicine, academic partnership, and health system reform in Ethiopia

**DOI:** 10.3389/fmed.2026.1897582

**Published:** 2026-09-28

**Authors:** Meseret Zerihun, Assegid Geleta, Katherine Dominique Rouleau, Praseedha Janakiram, Jamie Rodas, Marci Rose, Ellena Andoniou, Assefa abegaz, Dawit Wondimagegn

**Affiliations:** 1 Addis Ababa University, School of Medicine, Addis Ababa, Ethiopia; 2 Toronto Addis Ababa Academic Collaboration, Toronto, ON, Canada; 3 Department of Family and Community Medicine, University of Toronto, Toronto, ON, Canada; 4 Unity Health, St. Michael's Hospital, Toronto, ON, Canada; 5 The Institute for Education Research, Toronto, ON, Canada; 6 University Health Network (UHN), Toronto, ON, Canada; 7 The Ethiopia National Center of the African Health Observatory Platform on Health Systems and Policies, Addis Ababa, Ethiopia; 8 Addis Ababa University College of Health Sciences, Addis Ababa, Ethiopia

**Keywords:** Ethiopia, health profession education, health systems transformation, policy implementation, primary care workforce, primary health care (PHC), sub-Saharan Africa, Toronto Addis Ababa Academic Collaboration (TAAAC)

## Abstract

Since 2013, Ethiopia has trained more than 100 family physicians, a resource whose potential depends on integration of family medicine (FM) within a primary health care (PHC)-oriented health system. The Toronto Addis Ababa Academic Collaboration in Family Medicine (TAAAC-FM) has supported Ethiopia's inaugural FM residency since its inception. In 2025, TAAAC-FM co-convened a Policy Dialogue in Addis Ababa, with 40 participants from 10 countries to advance FM within PHC transformation. Five priorities emerged: FM identity and recognition; training reform; sustainable career pathways; health system alignment; equity and community engagement. Three recommendations: establishing government policy defining FM's role; expanding training and career pathways; and coordinating advocacy. Ethiopia's experience offers potentially relevant regional lessons for advancing FM toward Universal Health Coverage.

## 1 Introduction

### 1.1 Establishing family medicine as a specialty in Ethiopia

In 2013, Ethiopia launched its first family medicine (FM) residency programme, amid one of the world's most severe physician shortages (1), a physician-to-patient ratio of 1:36,158 (2), and some of the poorest health indicators globally (3, 4). Twelve years later, more than 100 family physicians have graduated, a second residency programme exists, and a growing continental movement has positioned FM as a cornerstone of PHC transformation across sub-Saharan Africa (SSA). Yet Ethiopia stands at a more demanding inflection point: the training infrastructure exists, political will is strengthening, and what remains is the harder work of embedding family physicians structurally within the health system. This brief argues that Ethiopia is ready for that phase, and its experience offers a model for the region.

This residency was not established in isolation. The Ethiopian Federal Ministry of Health (FMoH) had spent the preceding decade launching ambitious Health Sector Development Plans, including an unprecedented expansion of medical education between 2003 and 2009, during which universities and health science colleges surged from 5 to 23, and existing schools were tasked to triple or quadruple annual intake (1, 5). Emphasis was placed on expanding the physician workforce and postgraduate training (6). Within this context, FM emerged as a new specialty and driver of comprehensive, patient-centered, community-oriented primary care.

Responding to this policy imperative, and as part of a broader continental movement to build FM capacity across SSA (7, 8), the Toronto Addis Ababa Academic Collaboration in Family Medicine (TAAAC-FM) (9) was launched in 2013 (2). Grounded in global commitments to Primary Health Care (PHC) and Universal Health Coverage (UHC) (10), TAAAC-FM was designed not simply to train family physicians, but to build the educational, curricular, and leadership foundations a system-integrated specialty requires. The partnership links Addis Ababa University's (AAU) Department of Family Medicine with the Department of Family and Community Medicine (DFCM) at the University of Toronto (UofT), adapting a pre-existing psychiatry collaboration to the FM context with an explicitly Ethiopian-led orientation. What has emerged is a distinct postgraduate discipline, established at AAU, where most of Ethiopia's family physicians have trained and has recently extended to Saint Paul's Hospital Millennium Medical College (SPHMMC), home to the country's second FM residency built on AAU's expertise. While many institutions across Ethiopia continue to train general practitioners without the specialty-level, community-oriented foundation FM provides, these two programmes represent Ethiopia's entire FM graduate pipeline.

A hallmark of TAAAC-FM's approach is the sustained physical presence of visiting faculty. UofT faculty and senior residents travel to AAU for month-long visits several times annually, fostering the embedded engagement that underpins the collaboration's principles of respect, flexibility, cultural humility, and bidirectional learning (11). Reciprocally, AAU-FM faculty have co-led educational programmes, presentations, and leadership programming, including an AAU faculty fellowship year at UofT, and representation on a DFCM global policy and advocacy steering committee. TAAAC-FM has invested in the next generation of academic leaders on both sides, providing junior faculty and programme directors with mentoring, scholarship, and dissemination opportunities at their earliest career stages (11). AAU faculty overseeing curriculum design also hold influential positions in professional associations and governmental bodies shaping training policy, creating a direct pathway from academic partnership to policy influence and positioning AAU-FM faculty as participants in, rather than recipients of, the international FM conversation.

### 1.2 An unfinished policy agenda: from training to integration

As is common across many health systems undertaking rapid specialty development, growth in FM training capacity preceded the accompanying policy infrastructure. The primary gap lies between training family physicians and integrating them structurally within the health system. Residency training has remained predominantly hospital-based, and the specialty lacks a defined scope of practice and recognition outside academic circles. Meanwhile, financing structures privileging procedural specialisation have inadvertently marginalised FM, whose combination of generalist expertise, specialty-level training, and community-centered orientation remains undervalued and poorly rewarded.

These misalignments represent not a failure of the specialty, but an unfinished policy agenda this brief seeks to advance. Drawing on TAAAC-FM's longitudinal experience and a 2025 multi-country policy dialogue, it articulates a strategic pathway for establishing FM as a fully integrated driver of PHC transformation in Ethiopia, and a potential model for the region. Twelve years after TAAAC-FM's founding, conditions were ripe for a structured, multi-stakeholder dialogue to surface shared priorities: the April 2025 Policy Dialogue on “***Strengthening Primary Health Care through Family Medicine in Ethiopia.”***

### 1.3 Policy dialogue: design and methods

The Policy Dialogue was designed to catalyse strategic reflection on FM's integration into Ethiopia's PHC-oriented system, and identify actionable pathways for expanding the training, deployment, and recognition of family physicians as drivers of UHC, health equity, and resilient, community-centered care. On April 11, 2025, TAAAC-FM co-convened the dialogue in Addis Ababa, structured as a full-day programme of presentations, plenary discussion, and semi-structured breakouts. Forty participants, purposively selected for experience establishing or leading FM programmes, represented more than 15 governmental, non-governmental (NGO), academic, healthcare, financial, and professional organisations across 10 countries (Supplementary Table S1). FM residents and recent graduates ensured next-generation perspectives informed the dialogue.

Governance was deliberately Ethiopian-led, co-led by Dr. Dawit Wondimagegn and Professor Assefa Abegaz, with TAAAC-FM providing financial support and shared Ethiopian-Canadian facilitation, drawing on trusted relationships with WHO AFRO, JHPIEGO, and FM programmes across the continent. Several authors hold leadership roles within TAAAC-FM and Ethiopia's FM community; this insider perspective afforded longitudinal insight but could bias interpretation toward favourable views of the specialty and partnership. We sought to mitigate this through three independent note sets, two external frameworks (PHCMFI and WONCA) anchoring the synthesis, and manuscript co-development including authors not involved in facilitating the dialogue.

Discussion used a modified World Café methodology (12–14), with rapporteur notes as the primary data source. Participants deliberated in small groups in response to three guiding questions (Supplementary Box 1). Table report-backs captured breakout discussions within plenary themes; notes were taken concurrently by the plenary rapporteur and two TAAAC-FM leads, yielding three independent written records. Sessions were not audio-recorded. Rapid thematic synthesis across the three note sets identified recurring priorities and convergence; the five priorities were derived inductively. No formal coding was applied; triangulation relied on comparison across the note sets. A draft synthesis was reviewed and refined by Ethiopian and Canadian co-authors, all of whom had participated in the dialogue, providing collaborative validation across AAU, TAAAC-FM and partner institutions and ensuring alignment with dialogue content, policy context and implementation realities.

The dialogue and analysis were anchored in two complementary frameworks, which informed interpretation rather than serving as formal coding structures: the WHO Primary Health Care Measurement Framework and Indicators (PHCMFI) (15), which specifies what a PHC-oriented system must deliver and measure toward UHC, and the WONCA Core Principles of Family Medicine (16), which define the discipline and the competencies family physicians contribute. Supplementary Table S3 maps the five priorities against both frameworks. We acknowledge this approach's limitations. The dialogue was expert-driven: participants were purposively selected for FM leadership experience, a choice that enriched strategic insight but introduced selection bias toward those invested in its success. Patient and community voices were not directly represented, a limitation given this brief's equity focus. The community engagement mechanisms under Recommendations 1 and 3 partly address this gap; suggesting that future dialogue should include these representatives. The synthesis relied on rapporteur notes rather than transcribed, formally coded data, and reflects a pragmatic, practice-oriented analysis rather than formal qualitative research. Finally, the evidence supports the relevance and plausibility of the recommendations but does not establish their effectiveness; they are offered as policy options to be monitored using the indicators proposed (Supplementary Table S4).

Throughout, we distinguish findings emerging directly from the dialogue (the five priorities and the recommendations’ broad direction), claims supported by published evidence (cited), and the authors' interpretation, reflecting collective judgement rather than empirical demonstration.

## 2 Policy options and implications

### 2.1 Policy and system challenges

Despite policy recognition, FM faces structural barriers: undefined scopes of practice; inconsistent placement of family physicians in PHC leadership roles; limited academic departments and faculty; unclear career progression; and financing privileging specialist and hospital care. These misalignments contribute to attrition, underutilisation, and skepticism about FM's return on investment, yet they define precisely the agenda a system-integrated FM is positioned to address. Ethiopia stands at a policy window: a rare convergence of reform momentum, growing workforce capacity, and strengthened stakeholder consensus that creates genuine conditions for accelerating PHC transformation. The five priorities are presented as both diagnostic findings and strategic policy options (Supplementary Table S2).

#### 2.1.1 Strengthening the identity and recognition of family medicine

The misunderstanding of FM as a transitional stage toward sub-specialisation rather than a distinct, high-value discipline is well-documented across SSA, where uncertainties among policymakers about FM's role, placement, and value persist (17–19). In Ethiopia, misperception permeates medical education, professional culture, and public understanding, undermining recruitment and utilisation of family physicians. Addressing this requires defined national scopes of practice, strengthened FM departments, and professional and public education communicating the value of generalism as a lifelong career. Evidence from Botswana, Kenya, and Zambia suggests collaborative development of scope of practice between academic physicians and professional associations effectively increases awareness of the specialty's value (20).

#### 2.1.2 Reforming training models for real-world primary care

Current residency training remains predominantly hospital-centered, inadequately prepare graduates for the community-based, prevention-oriented, and relational practice PHC demands. Dialogue participants emphasised the urgent need for community-embedded “model family medicine clinics” as teaching and demonstration sites anchoring training in real-world primary care. In Ethiopia, most primary care is provided by health workers with limited clinical scope, driving excessive demand for specialist services and fragmented care (21). Residency training in FM, when anchored in community settings, is precisely positioned to address this gap (22). Sustainable reform requires investment not only in infrastructure but in a critical mass of locally trained academic family physicians who can drive curricular change from within (19, 23).

#### 2.1.3 Building sustainable career pathways for family physicians

Retention of family physicians, particularly in rural and underserved settings, remains a critical vulnerability. Weak professional pathways, urban migration, low relative remuneration, and limited advancement drive attrition. Approximately 73% of physicians leave the public sector for private hospitals, NGOs, or opportunities abroad (24, 25). Since centralised deployment was discontinued in 2019, hiring has devolved to regional health bureaus, leaving graduates navigating an inconsistent, decentralised landscape (26). For family physicians, deployment in primary care settings (principally health centres and primary hospitals) requires deliberate placement rather than market-driven absorption, since it is their sustained presence in these settings that enables the comprehensive, continuous coordinated care on which PHC-oriented systems depend. Sustainable retention requires rural practice allowances, academic career ladders, continuous professional development, and leadership pathways enabling contribution to governance and system design.

#### 2.1.4 Aligning health system structures to support primary care

Ethiopia's health system remains oriented toward hospital-centric, specialist-driven care, working against FM's integration into PHC delivery, that is, frontline, team-based promotive, preventive, curative, and rehabilitative services at health posts, health centres, and primary hospitals. Financing, governance, and service delivery must be reoriented to incentivise prevention, relational continuity, and community-based care. Critically, FM is not intended to replace existing cadres such as nurses, community health workers, midwives, and clinical officers, but to support, strengthen, and coordinate the services they already deliver. The consequences of these misalignments are already visible, highlighting a striking paradox: Ethiopia simultaneously faces one of the most acute physician shortages in SSA and a growing population of unemployed or underemployed medical graduates (24, 26, 27). This is not a supply failure, but a governance and financing failure that structured FM integration can help resolve (27).

#### 2.1.5 Advancing equity, social accountability, and community engagement

With more than 76% of its population being rural, Ethiopia is among the least urbanised countries in the world (28), and faces a complex, transitioning burden of communicable and non-communicable diseases (NCD), mental health conditions, and injuries (29–31), In this context, FM's community-rooted model is not simply preferable; it is an epidemiological necessity. Yet without a structured national matching system, physicians have gravitated toward urban centers, deepening rural-urban disparities (28, 32, 33). Equity-focused deployment frameworks, including rurally targeted incentives and coordinated placement, are essential to reaching the communities most in need, while social accountability mechanisms, including community engagement in governance and training design, keep the specialty responsive to the populations it serves.

### 2.2 Implications for PHC transformation

These priorities represent a coherent agenda for structural reform of how Ethiopia's health system trains, deploys, and supports its primary care physician workforce. Strong PHC-oriented systems can address up to 90% of common health needs (34, 35), and evidence from Brazil and Cuba demonstrates that a family physician on the primary care team contributes to effective, high-quality care (34, 36). A study in Rio de Janeiro found that FM residency training reduces hospitalisation and increases primary care follow-up (22). Systems with strong primary care foundations, characterised by first-contact access, continuity, comprehensiveness, coordination, and community orientation, achieve better outcomes, greater equity, and lower costs (37). Ethiopia's FM agenda is not merely a workforce initiative; it is a health system transformation strategy.

Academic institutions are critical to this transformation as engines of curricular innovation, community-linked service delivery, and policy-relevant research. University partnerships engage multiple stakeholders simultaneously, enabling multi-level capacity building across academia, government, NGOs, and civil society. Durable North-South partnerships such as TAAAC-FM demonstrate how specialty development can evolve beyond initial incubation toward sustainable, locally led transformation (11, 38). The dialogue's ten-country participation exemplifies the South-South learning networks that will sustain this work. For communities, FM offers more relational, preventive, and culturally responsive care. For the region, Ethiopia's experience offers an instructive example for countries navigating similar transitions.

## 3 Actionable recommendations

These recommendations are addressed to the full ecosystem of actors responsible for FM's integration: the FMoH, regional health bureaus, academic institutions, professional associations, and international partners. Supplementary Table S2 links each recommendation to the dialogue priorities and literature; Supplementary Table S4 specifies lead actors, actions, barriers, resources and monitoring indicators.

### 3.1 Recommendation 1: establish government policy defining the role of family physicians

Dialogue participants called on the FMoH, in collaboration with regional health bureaus, professional associations, medical councils, and the Ministry of Education, to formally adopt a national policy framework defining the scope of practice, placement, and governance role of family physicians within Ethiopia's PHC-oriented system. Building on a foundation that already exists the national specialty and sub-specialty roadmap identifies priority services for family physicians in primary hospitals, including chronic NCD, pediatric, and obstetrical/gynecological services, and acute care within a prevention framework (39). The recommended framework expands it. Concrete steps include a national scope of practice differentiating family physicians from general practitioners; positioning family physicians in well-equipped primary and district hospitals for team-based care; embedding FM within national and regional governance; aligning workforce strategies and UHC roadmaps to recognise FM's contribution; developing equity-focused deployment frameworks with rurally targeted incentives; and establishing a national FM research and monitoring agenda. Regional health bureaus are called upon to align hiring and budget allocation with this framework.

### 3.2 Recommendation 2: expand family medicine training and create sustainable career pathways

Ethiopia's growing FM workforce creates both opportunity and imperative for academic institutions, the FMoH, and international partners to expand and reform FM training, matching growth with the infrastructure, faculty capacity, and career pathways needed to retain and deploy graduates. Realising this potential, however, requires confronting significant barriers: inadequate funding, insufficient community-based training infrastructure, limited FM faculty, and constrained training opportunities (39). Priority actions: (1) rebuild where training happens: expanding FM departments at AAU and SPHMMC, building capacity at additional institutions, and anchoring longitudinal, community-based curricula in community-embedded model FM clinics doubling as demonstration sites; (2) sustain the pipeline: securing long-term funding and structured career pathways, from academic ladders and CPD to leadership development; and (3) track progress through a national FM workforce monitoring system. International partners, including TAAAC-FM, are called to continue supportive relationships while AAU-FM strengthens Ethiopian-led governance and delivery of training.

### 3.3 Recommendation 3: implement advocacy strategies for family medicine's integration

A collective commitment to amplifying FM's voice calls on professional associations, academic institutions, the FMoH, and international partners to sustain a coordinated advocacy strategy building the visibility, credibility, and political support necessary for integration. Key stakeholders, including policymakers, legislators, and the broader medical community, do not yet adequately understand FM as a distinct specialty, its added value, or return on investment. Actions include: equipping interest holders with evidence-based messaging on FM's added value, countering the misconception that comprehensive primary care requires no specialised training; engaging health financing institutions on models incentivising prevention and community-based care; engaging communities through governance mechanisms and patient advisory processes; leveraging regional networks (WONCA, AfroPHC, Primafamed) for South-South learning and advocacy; and documenting Ethiopia's FM journey as a continental example. TAAAC-FM and UofT's DFCM are called to continue supporting Ethiopian partners in knowledge translation and international advocacy.

## 4 Conclusions

Ethiopia's decade of investment in FM has laid a foundation few countries in the region can match — a growing cohort of family physicians, two residency programmes, and strengthened policy consensus that FM is essential to PHC transformation. The dialogue suggested the conditions for the next phase are in place: reform momentum, regional solidarity, and a generation of FM leaders ready to carry the work forward. What is required now is political will and structural investment to translate momentum into lasting change through clear policy frameworks, reformed training, and sustainable career structures. These challenges are not unique to Ethiopia. Across SSA, countries building FM programmes have confronted the same tensions between training capacity and system integration, specialist-driven financing and generalist-oriented care, and between international partnership and local ownership. Ethiopia's experience shows these tensions can be navigated. Outputs from the dialogue and the May 2025 Starfield Summit reinforce a continental consensus: professional identity must be championed, FM leaders must hold positions of influence within associations and governance structures, and advocacy must be sustained across political cycles and institutional transitions.

The dialogue's most powerful message was generational responsibility, a call for residents, new graduates, and early-career physicians to step forward, take ownership of the discipline's future, and ensure that the momentum of the past decade does not dissipate. This brief is a contribution to that effort. The pathway it outlines is not a distant aspiration, but an actionable agenda, grounded in evidence and stakeholder consensus, that Ethiopia's health system actors can begin to implement now.
