# Supplementary material for: Building primary health care from the ground up: family medicine, academic partnership, and health system reform in Ethiopia
Source: Front Med (Lausanne). 2026 Sep 28;13:1897582. doi: 10.3389/fmed.2026.1897582 (PMC13654837; doi:10.3389/fmed.2026.1897582)
Supplement: Supplementary file 1 [file Datasheet1.pdf]

Supplementary Box 1: Key questions used to guide the World Café discussion

- How might family medicine be integrated in the Ethiopian health system to maximise its impact on PHC performance for UHC, including in enabling the achievement of high-quality primary care (and its 5Cs) at the core of integrated health services?

- What advocacy and information strategies are needed to improve knowledge about, and increase the perceived value of, family medicine in Ethiopia for key stakeholders including government leaders, policy makers, other health professionals and health workers, their professional associations, and the population?

- What role and scope should family physicians have in Ethiopia to best support primary health care-oriented health systems generally, and enable the delivery of high-quality primary care services more specifically?

Note: Questions are reproduced from the dialogue facilitation materials shared with all participants prior to the policy dialogue. The "5Cs" refer to the core attributes of high-quality primary care: first-contact access, continuity, comprehensiveness, coordination, and community orientation.

Supplementary Table 1: Geographic and institutional representation among interest holders

Category	Representation
Countries represented	Ethiopia, Uganda, Rwanda, Kenya, Madagascar, Angola, Senegal, Nigeria, Brazil, and Canada
Government and public-sector institutions	Ethiopian Federal Ministry of Health (FMoH); Ethiopian Regional Health Bureaus; Development Bank of Ethiopia
Multilateral and regional health organisations	WHO African Health Observatory Platform on Health Systems and Policies (AHOP); WHO Regional Office for Africa (AFRO); African Forum for Primary Health Care (AfroPHC); World Organisation of Family Doctors (WONCA)
Academic and training institutions	Addis Ababa University School of Medicine; Addis Ababa University Department of Family Medicine; Saint Paul's Hospital Millennium Medical College; University of Toronto Department of Family and Community Medicine
Clinical institutions	Tikur Anbessa Specialised Hospital
Professional associations and implementation organisations	Ethiopian Association of Family Medicine Specialists (AEFMS); JHPIEGO
TAAAC-FM leadership	Directors, Co-Chair, National Lead, and Programme Leads from Ethiopia and Canada

Supplementary Table 2: Mapping of the five dialogue priorities to illustrative findings, supporting literature, and linked recommendations. Detailed alignment of each priority with the PHCMFI and WONCA frameworks is provided in Supplementary Table 3.

Priority	Illustrative dialogue findings	Supporting literature (refs)	Linked recommendation(s)	Framework anchor
1. Identity & recognition of FM	FM misunderstood as a transitional stage toward sub-specialisation; absence of a defined national scope of practice undermines recruitment and utilisation; need for professional and public education on the value of generalism	(17–20)	Rec 1 (scope of practice); Rec 3 (advocacy)	WONCA core competencies; PHCMFI workforce capacity
2. Training models for real-world primary care	Training remains hospital-centred; urgent call for community-embedded model FM clinics as teaching and demonstration sites; need for locally trained academic FPs to drive curricular change	(19, 21–23)	Rec 2 (training expansion & reform)	PHCMFI service delivery; WONCA community orientation
3. Sustainable career pathways	Attrition driven by weak pathways, low relative remuneration, urban migration; decentralised hiring leaves no deliberate placement mechanism for FPs in primary care settings	(24–26)	Rec 2 (career pathways); Rec 1 (deployment frameworks)	PHCMFI health workforce; WONCA person-centred continuity
4. Health system alignment	Hospital-centric, specialist-driven financing and governance work against FM integration; coexistence of physician shortage and unemployed graduates reflects governance and financing failure	(24, 26, 27)	Rec 1 (governance embedding); Rec 3 (financing advocacy)	PHCMFI governance & financing
5. Equity, social accountability, community engagement	Predominantly rural population with a complex burden of disease faces deepening rural–urban maldistribution; equity-focused deployment and community engagement mechanisms needed	(28–33)	Rec 1 (equity-focused deployment); Rec 3 (community engagement)	PHCMFI equity & access; WONCA social accountability

Supplementary Table 3: Mapping the five dialogue priorities onto the WHO Primary Health Care Measurement Framework and Indicators (PHCMFI) and the WONCA Core Principles of Family Medicine

The five priorities were derived inductively from rapporteur notes; the mapping below was undertaken retrospectively by the authorship team to demonstrate how the emergent themes align with the two sensitising frameworks. PHCMFI domains follow the framework's structure of health system determinants, service delivery, and performance; WONCA principles follow the core competencies and characteristics of the discipline as defined in the European Definition of General Practice/Family Medicine.

Dialogue Priority	Relevant PHCMFI Domains	Relevant WONCA Core Principles	Rationale for Mapping
1. Strengthening the identity and recognition of family medicine	Governance (PHC policies, leadership and workforce planning); Health workforce (competencies and role definition)	Definition of the discipline as a distinct specialty; specific problem-solving skills (decision-making based on community prevalence and incidence); person-centred care	Recognition of FM requires governance-level clarity on scope of practice (PHCMFI governance domain) and a shared understanding of the distinct competencies that differentiate family physicians from non-specialist generalists (WONCA disciplinary definition)
2. Reforming training models for real-world primary care	Health workforce (education and training aligned to service delivery needs); Models of care (community-based, people-centred service organisation); Quality of care	Comprehensive approach (managing acute and chronic conditions, prevention, and promotion); community orientation; holistic approach (bio-psycho-social model)	Hospital-centered training misaligns workforce preparation with the community-embedded models of care the PHCMFI specifies; WONCA's comprehensiveness and community orientation principles define the competencies such training must produce
3. Building sustainable career pathways for family physicians	Health workforce (availability, distribution, retention); Health financing (remuneration and incentive structures)	Continuity of care (longitudinal, relational continuity depends on workforce stability); person-centred care	Retention and equitable distribution are explicit PHCMFI workforce indicators; WONCA's continuity principle can only be realised where career structures keep family physicians in sustained community practice
4. Aligning health system structures to support primary care	Governance; Health financing (allocation toward PHC); Models of care (integration and referral systems); Physical infrastructure	Primary care management (first-contact access, coordination of care, gatekeeping and navigation functions)	System structures that privilege hospital-centric care undermine the first-contact and coordination functions WONCA assigns to FM; the PHCMFI's governance, financing, and models-of-care domains identify the structural levers requiring realignment

Dialogue Priority	Relevant PHCMFI Domains	Relevant WONCA Core Principles	Rationale for Mapping
5. Advancing equity, social accountability, and community engagement	Community engagement (empowered people and communities as a core PHC component); Equity (performance domain); Access and effective service coverage	Community orientation; holistic approach; person-centred care responsive to population context	Equity and community empowerment are both a foundational PHC component and a measured performance outcome in the PHCMFI; WONCA's community orientation principle positions family physicians as accountable to defined populations, linking disciplinary practice to system-level equity goals

Note: The PHCMFI and WONCA frameworks operate at complementary levels of analysis: the PHCMFI at the level of health system capacity, service delivery, and measurable performance toward UHC, and the WONCA principles at the level of the discipline and the competencies of its practitioners. Several priorities therefore map to both frameworks simultaneously, reflecting the dialogue's central argument that family medicine integration is both a workforce question and a system design question.

Supplementary Table 4: Implementation matrix for the three recommendations, specifying lead actors, sequenced actions, potential barriers, resource implications, and indicators for monitoring progress.

Recommendation	Lead actors	Short-term actions (1–2 y)	Medium-term actions (3–5 y)	Potential barriers & resources	Indicators of progress
1. National policy framework defining FM's role	FMoH (lead); regional health bureaus; medical councils; professional associations; Ministry of Education	Draft national FM scope of practice differentiating FPs from GPs; convene interest-holder consultations; embed FM focal points in governance structures	Formally adopt policy framework; align workforce strategy and UHC roadmap; implement equity-focused deployment framework with rural incentives	Specialist-dominated governance; decentralised authority across regions. Resources: modest — policy development and consultation costs	National scope of practice adopted; number of FPs deployed in primary and district hospitals; FM named in national workforce strategy and UHC roadmap
2. Expand training & create career pathways	AAU & SPHMMC (lead); FMoH; regional health bureaus; international partners incl. TAAAC-FM	Establish first community-embedded model FM clinics; begin shift of residency curricula toward longitudinal community-based learning; design academic career ladders	Expand FM departments and add training sites; institutionalise CPD frameworks and leadership pathways; launch national FM workforce monitoring system	Funding constraints; limited FM faculty; training infrastructure gaps. Resources: substantial — infrastructure, faculty posts, rural allowances	Number of residency positions and graduates per year; proportion of training time in community settings; public-sector retention rate of FPs at 3 and 5 years
3. Coordinated advocacy for FM integration	Professional associations (AEFMS, lead); academic institutions; FMoH; WONCA, AfroPHC, Primafamed networks	Develop advocacy strategy with evidence-based messaging on FM's value and return on investment; targeted engagement of legislators and financing institutions	Institutionalise community advisory processes in FM governance and training design; sustain South-South dissemination of Ethiopia's FM experience	Persistent misconceptions about generalism; political and institutional turnover. Resources: modest — coordination and communication costs	Documented advocacy strategy; number of financing dialogues held; community advisory mechanisms established; FM referenced in policy documents
